# Gamma Tocopherol and Lovastatin Additively Induced Apoptosis in Human Colorectal Carcinoma Cell Line (HT29)

**Published:** 2012-10-07

**Authors:** Leila Zeidooni, Mohsen Rezaei, Mahmood Hashemi Tabar

**Affiliations:** 1 Department of Toxicology, Ahvaz Jundishapur University of Medical Sciences, Ahvaz, IR Iran; 2 Department of Cellular and Molecular Research Center, Ahvaz Jundishapur University of Medical Sciences, Ahvaz, IR Iran

**Keywords:** Apoptosis, Lovastatin, gamma-Tocopherol, Flow Cytometry

## Abstract

**Background:**

Programmed cell death (apoptosis) is a physiological process needed to remove unwanted or damaged cells. It has been hypothesized that any failure of programmed cell death leads to the development of neoplasm. Identifying new agents which induce apoptosis in tumor cells is of great significance in treatment of neoplasms. Numerous studies suggest that exposure of tumor cells to statins and gamma tocopherol can lead to cell death.

**Objectives:**

The aim of this study was to evaluate the cell death induced by gamma tocopherol and lovastatin in human colorectal carcinoma cell line (HT29) using flow cytometry.

**Material and Methods:**

HT29 cells were grown in DMEM medium, exposed to different concentrations of lovastatin (10,20,40,100μM ) and gamma tocopherol (25,50,100,200μM) for 48 and 72 hours, individually and in combination (100μM both, 48 h). Phenotype of apoptosis was determined by means of flow cytometry.

**Results:**

All Concentrations of lovastatin (10, 20, 40, 100 μM) and gamma tocopherol (25, 50, 100, 200 μM) induced an apoptotic response in HT29 cells. In combination, a significant increase in apoptosis phenotype was also demonstrated (P < 0.05).

**Conclusions:**

This study showed that lovastatin when combined with gamma tocopherol, could induce apoptosis in HT29 cells more potently than each agent alone, which uncovers the significance of targeting the proliferative signaling in different points of the pathway.

## 1. Background

Cancer is one of the leading causes of mortality. Colorectal cancer is the second most common cause of cancer deaths in the United States for both sexes. In western countries, lifetime risk of developing colorectal cancer in the general population approximates 5% (1). Some studies have established that COX-2 (cyclooxygenase-2) over expression is common to a variety of human malignancies, including cancer of the colon, also it promotes tumor cell growth, angiogenesis, tumor invasion, and metastasis (2-4). In colon carcinogenesis, COX-2 is elevated in premalignant lesions which remains at very high levels as tumors progress to a malignant neoplasm (5). Findings have shown that gamma tocopherol (γ-T) and its metabolite γ-CEHC possess anti-inflammatory properties (COX-2 inhibition) and various animal and human tumor tissues, including human colon cancer, have been reported to contain increased COX-2 expression and PGE2 that PGE2 has been shown to promote proliferation in certain cancer cells (6). Different studies suggest that the risk of human colon cancer is increased by the mutagenic actions of free radicals, which are produced during oxidation reactions. Dietary antioxidants, such as vitamin E, is intended to reduce the levels of these harmful oxidative products (7). Lovastatin, (HMG-CoA reductase inhibitor), which blocks the synthesis of cholesterol, has been reported to induce apoptosis in cancer cells (8). While programmed cell death, or apoptosis, is important for the development and homeostasis of tissues, abnormal cell death can result in some autoimmune diseases or may be cancer (9). Colorectal carcinogenesis is related to the inhibition of apoptosis and the augmentation of proliferative activity probably by accumulation of genetic mutations (10). Apoptotic cells have many characteristics that can be detected with flow cytometry. These characteristics include cell plasma membrane rearrangements, changes in plasma membrane and mitochondrial membrane permeability, caspases activation, and DNA cleavage. Determination of these alterations by flow cytometry allows the identification and quantification of apoptotic cells (11). Lovastatin has induced apoptosis in ovarian cancer cells in a p53-independent manner (12). Tocopherols are lipid soluble antioxidants which exist as eight structurally isoforms (13). γ-T is the most prevalent form of vitamin E in plant seeds and products derived from these food staff (14). γ-T is a potent pro-apoptotic agent for human breast cancer cells and apparently induces apoptosis by activation of DR5-mediated apoptotic pathway (15). Statins in clinical doses reduce serum cholesterol levels and lower the incidence of myocardial infarction. However in high doses which is necessary for apoptosis induction may be associated with significant toxicity. Radiation or chemotherapy, are often effective in causing remission, however lead to deleterious side effects. It is therefore important to develop effective anticancer agents with high selectivity for malignant cells and low toxicity. Thus to reduce the toxicity and also for maintain chemopreventive strategies, and based on our previous findings, it seems that statins when used simultaneously with other relevant agents can provide additive cytotoxic effects (16, 17). We suggest that gamma tocopherol and lovastatin in combination may fall into this category and be beneficial for human cancer prevention.

## 2. Objectives

The aim of this study was to investigate the effect of γ-T and lovastatin individually and simultaneously on programmed cell death induction in human colorectal carcinoma cell line (HT29) using flow cytometry.

## 3. Materials and Methods

### 3.1. Materials

Cell culture materials including Dulbecco`s Modified Eagle Medium (DMEM) were obtained from Applichem, Penicillin-Stereptomycin, trypsin, and fetal bovine serum were obtained from Gibco, Dimethyl sulfoxide(DMSO), Gamma tocopherol, Anisomycin, and phosphate buffer saline(Nacl, Kcl, Na_2_HPO_4_(2H_2_O), KH_2_PO_4_) were obtained from Sigma Chemicals, Lovastatin was purchased from Biocon, HT29 cell line was obtained from Pasture Institute, Tehran Iran, FLOWCellect^TM^ , Annexin Red Kit was purchased from Millipore Co.

### 3.2. Preparation of Drugs

25mg of γ-T was dissolved in 10 ml of DMSO. Then different concentrations (25, 50, 100, 200µM), were prepared using the stock solution. For lovastatin activation, 40mg lovastatin was dissolved in 1ml ethanol (96%), heated to 50°C, then alkalinized by adding 1.5 ml NaOH(1M), followed by 2h incubation, after that HCL was added to neutralize the solution, and finally stored frozen at -20°C.

### 3.3. Cell Culture

HT29 human colon cancer cell line grown in Dulbecco’s Modified Eagle Medium with L-glutamine, and 10% fetal bovine serum (FBS), penicillin G (100 U/mL), streptomycin (100 U/mL) and in a humidified atmosphere of 5% CO2 at 37°C. HT29 human colon cancer cells were seeded at a concentration of 1 × 10^6^ in flasks and viability of cells was determined using trypan blue staining, cells were exposed to γ-T (25µM, 50µM, 100µM, and 200µM), and Lovstatin (10µM, 20µM, 40µM, and 100µM) for 48 and 72 hours, individually and in combination (100µM, 48 h).

### 3.4. Evaluation of Apoptosis by Annexin V and 7-Aminoactinomycin D (7-AAD) Staining

After incubation of HT29 cells with different doses of γ-T and lovastatin for 48 and 72h, both floating and attached cells were collected by trypsinization and then cells were stained with annexin V, CF647-7/AAD. Firstly cells were co-incubated with CF647-labeled annexin V as an early marker of apoptosis, and subsequently coincubated with 7-AAD as a late marker of apoptosis; cells were quantified using flow cytometry FACSCalibure (Becton Dickinson) to differentiate between early apoptosis and late apoptosis. Positive control group received 2µg/ml/2h anisomycin and cells in negative control group (untreated group) did not expose to any agent. The externalization of phosphatidyl serine from plasma membrane, a marker of apoptosis, is recognized by annexin V conjugated with fluorescein CF647 and 7/AAD penetrates into the plasma membrane of cells that have lost membrane integrity.

### 3.5. Statistical Analysis

All experiments were performed in at least three trials. Student’s T test was applied to determine statistical significance with a P value lower than0.05. ANOVA test was used when more than two groups were compared.

## 4. Results

**Figure 1:**
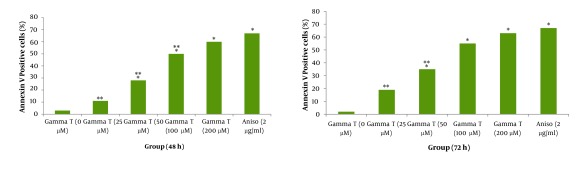
Induction of apoptosis with various concentrations of γ-T (0, 25, 50, 100 and 200μM) using annexin V–CF647 staining and flow cytometry in HT29 cell line for 48h (A) and 72h (B). HT29 cells were grown in DMEM, exposed to specific agents and early apoptosis was determined by annexin V staining method. Cells in positive control group received 2μg/ml/2h anisomycin * Significant difference in comparison with untreated group (P < 0.05).** Significant difference in comparison with anisomycin treated group (P < 0.05)

**Figure 2:**
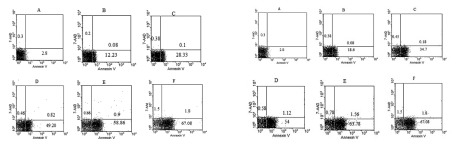
Dot plot view for induction of apoptosis with various concentrations of γ-T (0, 25, 50, 100 and 200μM) using annexin V– CF647/7-AAD staining and flow cytometry in HT29 cell line for 48h and 72h. CF647-labeled annexin V was an early marker for phosphatidyl serine externalization over cell membrane (lower right quadrant) and late apoptosis was labeled with 7-AAD in the upper left quadrant. HT29 cells were grown in DMEM, exposed to specific agents and early apoptosis was determined by annexin V staining method. Cells in positive control group received 2μg/ml/2h anisomycin. A) Untreated cells. B, C, D and E are groups that received 25, 50, 100 and 200μM γ-T. F) Treated cells with anisomycin 2μgr/ml/2h.

**Figure 3:**
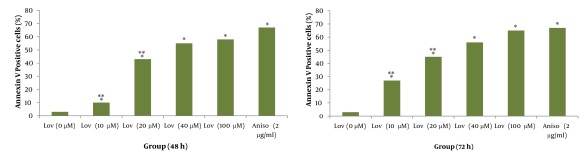
Induction of apoptosis with various concentrations of lovastatin (0, 10, 20, 40 and 100μM) using annexin V–CF647 staining and flow cytometry in HT29 cell line for 48h (A) and 72h (B). HT29 cells were grown in DMEM, exposed to specific agents and early apoptosis was determined by annexin V staining method. Cells in positive control group received 2μg/ml/2h anisomycin. * Significant difference in comparison with untreated group (P < 0.05).** Significant difference in comparison with anisomycin treated group (P < 0.05).

**Figure 4:**
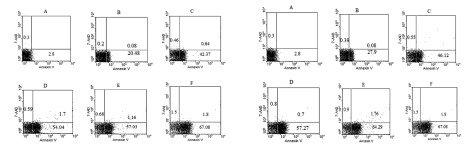
Dot plot view for induction of apoptosis with various concentrations of lovastatin (0, 10, 20, 40 and 100μM) using annexin V– CF647/7-AAD staining and flow cytometry in HT29 cell line for 48h and 72h. CF647-labeled annexin V was an early marker for phosphatidyl serine externalization over cell membrane (lower right quadrant) and late apoptosis was labeled with 7-AAD in the upper left quadrant. HT29 cells were grown in DMEM, exposed to specific agents and early apoptosis was determined by annexin V staining method. Cells in positive control group received 2μg/ml/2h anisomycin. A Untreated cells. B, C, D and E are groups that received 10, 20, 40 and 100μM γ-T. F Treated cells with anisomycin 2μgr/ml/2h.

As shown in Figures 1 and 2 annexin V positive cells (early apoptosis) were significantly higher in groups received 50, 100 and 200µM γ-T compared with control groups after 48 and 72 hours. As seen γ-T concentrations of 200µM for 48h and also 100 µM and 200 µM after 72h could not induce apoptosis as much as the positive control group (anisomycin 2µg/ml/2h). As shown in Figures 3 and 4 annexin V positive cells (early apoptosis) were significantly higher in groups received 10, 20, 40 and 100µM lovastatin compared with control group after 48 and 72 hours. Lovastatin concentrations of 40 and 100µM for 48h and also for 72h could not induce apoptosis as much as the positive control group (anisomycin 2µg/ml/2h).

**Figure 5:**
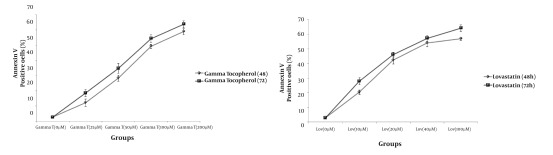
Annexin V positive cells (early apoptosis) determined by annexinV–CF647 staining and flow cytometry in HT29 cell line with various concentrations of γ-T (A), Lovastatin (B) for 48h and 72h. HT29 cells were grown in DMEM, exposed to specific agents and early apoptosis was determined by annexin V staining method.

**Figure 6:**
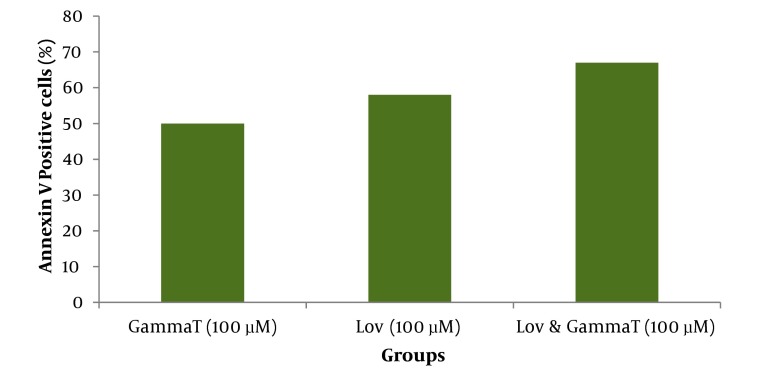
Annexin V positive cells (early apoptosis) determined by annexinV– CF647 staining and flow cytometry in HT29 cell line. HT29 cells were grown in DMEM, exposed to specific agents and early apoptosis was determined by annexin V staining method. Far right group received 100μM γ-T and also lovastatin for 48h simultaneously. * significant difference compared with γ-T (100μM, 48h) and lovastatin (100μM, 48h) groups individually.

**Figure 7:**
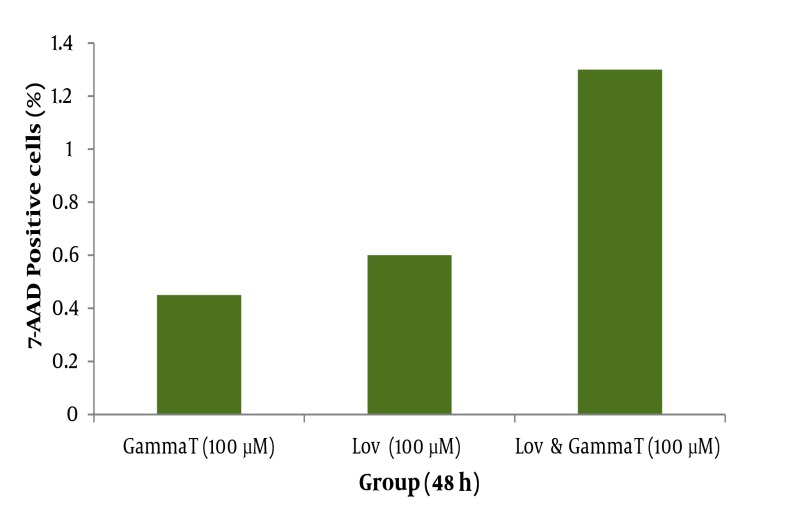
Late apoptosis positive cells determined by 7-AAD staining and flow cytometry in HT29 cell line. HT29 cells were grown in DMEM, exposed to specific agents and late apoptosis was determined by 7-AAD staining method. Far right group received 100μM γ-T and also lovastatin for 48h simultaneously. * significant difference compared with γ-T (100μM,48h) and lovastatin (100μM, 48h) groups individually.

As demonstrated in Figure 5, there is no significant difference (P < 0.05) for apoptosis induced by various concentrations of γ-T and lovastatin in 48h compared with the results obtained for 72h. Figures 6 and 7 show the combination effect of γ-T and lovastatin on apoptosis induction and it is clear that these two agents simultaneously (100µM for both, 48h), lead to more powerful effects on apoptosis induction. Late apoptosis positive cells (Figure 7) determined by 7-AAD staining and positive cells determined by annexinV–CF647 staining and flow cytometry in HT29 cell line represent early apoptosis. Results of 7-AAD staining in different groups were similar to annexin V results and have not been expressed accordingly.

## 5. Discussion

Effective chemoprevention strategies for colorectal cancer can be focused on promotion or restoring apoptosis (18). While individual agents may inhibit proliferation and induce apoptosis in high doses, but toxic effects could be ensued. One way to decrease these toxic side effects and also increase the effectiveness of treatment is to employ combination regimens that each component individually has shown good activity against tumor cells. This will provide us a relevant tool to reduce the dose of each agent. On the other hand, tumor cells resistance will be decreased when the activation of cell death pathway targeted by relevant chemicals with different mechanisms of action. As mentioned previously, γ-T and lovastatin are good candidates for this approach of treatment.

Based on previous studies, we prepared 10, 20, 40, and 100µM (48 h) of lovastatin concentrations, by which all groups showed significantly higher apoptosis levelscompared with control groups (Figures 3 and 4). The most apoptotic response was obtained by 100µM of lovastatin .Therefore with increasing the concentration of lovastatin, apoptotic phenotype showed a dose dependent response. Also, after a 72-hour exposure of HT29 cells to different lovastatin concentrations the same results were obtained and accordingly a dose dependent manner was obvious (Figures 3 and 4). On the other hand, comparison of data achieved from 72 and 48 hours exposure of HT29 cells to different concentrations of lovastatin, revealed no time dependent relationship for apoptosis induction (Figure 5). Many studies showed the effectiveness of statins against tumors. Martirosyan et al (2010), found that ovarian cancer cells are sensitive to statin induced apoptosis and strongly suggested that statins can play a role in the treatment of ovarian carcinoma (12). Padayatty S J et al. (1997), found that lovastatin induces apoptosis in cultured prostate stromal cells (19). Many studies have shown that lovastatin induce apoptosis in hepatocytes (20), lymphocytes (21), leukemia cells (22), and prostate cancer cells (19) in culture.

Effect of γ-T (25, 50, 100, 200 µM) alone on apoptotic response of HT29 cells, was determined previously, therefore we investigated an additive effect of γ-T to lovastatin. Firstly, cells were incubated for 48 hours with different γ-T concentrations (50, 100, 200 µM) and a dose dependent pattern likewise lovastatin effect has been achieved (Figure 1, 2 and 5). These results confirmed by numerous previous works. Gysin et al. (2002) showed that γ-T inhibits proliferation of prostate and colon cancer cells (23). Jiang Q et al. (2003), showed that γ-T and its major metabolite, γ-CEHC, significantly inhibited pro inflammatory eicosanoids at the site of inflammation (24). Following combination, our results showed that when cells treated with γ-T and lovastatin simultaneously, early and also late apoptotic events revealed a significant increase compared with control groups(P < 0.05) (Figure 6). In all groups, cell death increased in a dose dependent manner, however, our data demonstrated that there is no time dependency among treated groups. Thus the cell death mechanism induced together, appeared to be triggered from the mitochondria. This notion was recently searched in our lab, and data (not shown) revealed mitochondrial involvement in cell death induced by statin and γ-T.

Therefore statins and γ-T seems to be more powerful when used simultaneously with other effective agents against tumors. Such data are now presented: Swamy M V et al. 2002, showed effectiveness of lovastatin and celecoxib (a cyclooxygenase-2 inhibitor), individually or in combination on lamin B levels, caspase-3 activity and apoptosis in human HT29 colon cancer cell. Lamin B levels were significantly increased in a dose-dependent manner in cells treated with lovastatin but no such effect was observed with celecoxib, but in combination, synergistically enhanced caspase-3 activities (25). Agarwal B et al 1999, showed that lovastatin augments apoptosis at 10 µM and 30µM concentrations concomitant to standard chemotherapeutic agents such as 5-FU and cisplatin in colon cancer cells (26).

Altogether our study demonstrates that γ-T and lovastatin induce apoptosis in HT29 cell line (P < 0.05). The combination of both, exhibit additive or may synergistic effects (P < 0.05). This study suggests the usefulness of lovastatin and γ-T as an adjuvant to current chemotherapeutics for colorectal cancer patients, and demonstrating that combination treatments may have clinical applications.
